# Herpes Simplex Virus Type 1 Interactions with the Interferon System

**DOI:** 10.3390/ijms21145150

**Published:** 2020-07-21

**Authors:** Kevin Danastas, Monica Miranda-Saksena, Anthony L. Cunningham

**Affiliations:** Centre for Virus Research, The Westmead Institute for Medical Research, The University of Sydney, Westmead NSW 2145, Australia; kevin.danastas@sydney.edu.au (K.D.); monica.saksena@sydney.edu.au (M.M.-S.)

**Keywords:** herpes simplex virus, interferon, innate immunity, immune evasion

## Abstract

The interferon (IFN) system is one of the first lines of defense activated against invading viral pathogens. Upon secretion, IFNs activate a signaling cascade resulting in the production of several interferon stimulated genes (ISGs), which work to limit viral replication and establish an overall anti-viral state. Herpes simplex virus type 1 is a ubiquitous human pathogen that has evolved to downregulate the IFN response and establish lifelong latent infection in sensory neurons of the host. This review will focus on the mechanisms by which the host innate immune system detects invading HSV-1 virions, the subsequent IFN response generated to limit viral infection, and the evasion strategies developed by HSV-1 to evade the immune system and establish latency in the host.

## 1. Introduction

The innate immune system is the first line of defense against invading pathogens. Perhaps the most widely studied aspect of innate immunity is the interferon (IFN) response. IFNs are a family of cytokines secreted by several cell types upon the detection of an invading pathogen, particularly viruses. The IFN response is important in clearing the virus during acute infections and establishing an anti-viral state. However, certain families of viruses, such as the Herpesviruses, have evolved to establish lifelong latent infections without being detrimental to the health of the host. The innate immune system, including IFN, is important in maintaining a balance between host and virus to prevent disease and death [1]. This review aims to discuss the innate immune response generated in response to Herpes Simplex Virus (HSV) type 1 infections, and the evasion mechanisms that the virus has developed to persist and establish life-long latent infections in the host.

## 2. Herpes Simplex Virus

The family of Herpesviruses contains over one hundred viruses, nine of which are able to cause disease in humans. Herpesviruses are divided into three subfamilies, alpha-, beta- and gammaherpesviruses, based on the viral genome, viral characteristics, and the cell type where latency is established [1,2]. Alphaherpesviruses are defined by their ability to establish latency in neurons, and include the human tropic viruses, HSV-1, HSV-2, and varicella-zoster virus (VZV) [2,3]. HSV-1 is prevalent worldwide, is estimated to infect 45–90% of the world’s population, and is highest in the developing world [3].

HSV-1 is approximately 225 nm in diameter and is made up of four components: a DNA core, capsid, tegument, and envelope. The core contains the linear double stranded DNA (dsDNA) genome (152 kb), which is surrounded by an icosahedral capsid consisting of 6 proteins. This nucleocapsid is surrounded by a dense proteinaceous matrix called the tegument, which consists of 23 tegument proteins. The tegument then links to and is enclosed in a host-derived lipid bi-layer called the envelope, studded with 10 viral glycoproteins [4,5,6].

Primary infections of HSV-1 are usually established in the oral mucosa, and less frequently in the genital mucosa or other epithelial surfaces at the periphery. While HSV-2 is the main causative agent of genital herpes overall, the main cause of initial genital herpes is HSV-1 in populations such as young women [7,8,9,10]. Following infection and replication in stratified squamous epithelial cells, the virus infects innervating sensory nerves in the epidermis and undergoes retrograde axonal transport to the cell body. Here, the viral genome is deposited in the nucleus establishing a life-long latent infection primarily in the trigeminal ganglia (TG) or dorsal root ganglia (DRG), but may be present in other sensory or sympathetic ganglia, such as the superior cervical ganglia, depending on the original site of infection [11,12]. In neurons, the viral genome is silenced, inducing a latent state tightly controlled by host cellular mechanisms [13]. During this state of latency, the virus is largely transcriptionally silent, with the exception of a single transcript known as the latency-associated transcript (LAT) [14]. LATs are thought to be involved in inhibiting cellular apoptosis through several mechanisms, including the downregulation of IFNs [15]. HSV-1 can undergo sporadic reactivation and begin replicating, with viral particles and proteins undergoing anterograde axonal transport back to the initial site of infection where they are released from the nerve endings to epithelial cells, where viral replication occurs [16,17,18]. This then results in either herpes lesions, or more commonly, asymptomatic shedding. The extent and frequency of lesions depends partly on viral and host genetics, the latter through immune control [19]. Clinical manifestations of HSV-1 infections in immunocompetent hosts most commonly include minor orofacial lesions (commonly known as cold sores), but can present as genital herpes, keratitis, and peripheral skin lesions [14]. In rare cases, complications and serious illness can arise, particularly in neonates or the immunocompromised. HSV-1 can travel to the central nervous system, resulting in herpes encephalitis (HSE) or circulation to cause disseminated herpes [20,21].

### Entry, Replication, and Release of HSV-1

Viral attachment and entry into cells are mediated by the interaction of viral glycoproteins with cellular membrane receptors. Initial binding occurs through envelope glycoprotein C (gC) and/or gB binding to heparin sulfate proteoglycans, allowing gD to then bind to one of three receptors to initiate viral entry; herpesvirus entry mediator (HVEM), nectin-1, and 3-O sulfated heparin sulfate [22,23,24]. The receptor involved is cell type dependent, e.g., nectin-1 is the main receptor for epithelial cells, neuronal cells, and fibroblasts [25,26,27,28,29], whereas HVEM is the main receptor for T-cells and corneal epithelial cells [24,30]. However, there is evidence that the loss of one receptor can be compensated for by another [26]. Regardless of the receptor used, upon binding, gD undergoes a conformational change allowing gB, aided by the heterodimer gH/gL, to initiate fusion of the virus to the plasma membrane [31]. Depending on the cell type, this fusion can either be pH-dependent (e.g., epidermal keratinocytes) after clathrin dependent endocytosis, or pH-independent usually at the cell membrane (e.g., neurons) [32,33]. Following entry, there is the loss of the viral envelope proteins, as well as most tegument proteins, and the resulting nucleocapsid (now only surrounded by the inner tegument proteins unique short (US) 3 (pUS3), unique long (UL) 36 (pUL36) and pUL37) is transported along microtubules to the nucleus, where the genome is deposited and replication takes place [6,14].

Following replication, the nucleocapsid is assembled within the nucleus, and undergoes primary envelopment-de-envelopment as it passes through the nuclear membrane, releasing the naked nucleocapsid into the cytoplasm. Here in the cytoplasm, the naked nucleocapsid acquires inner tegument proteins by invaginating vesicles derived from the *trans*-Golgi network, acquires outer tegument proteins, as well as undergoing secondary envelopment. The fully enveloped virus is then transported within a vesicle to the plasma membrane for final release [6,14]. While this process occurs in most cell types, a different pathway is undertaken for virus entry, transport, and release from the axons of sensory neuronal cells. For a detailed review of this process, please see [6].

## 3. Recognition of HSV-1 by the Innate Immune System

### 3.1. Introduction

Throughout the viral life cycle, there are several stages where the innate immune system can detect the presence of HSV-1 and upon recognition, mount an immune response to clear the virus. Pattern recognition receptors (PRRs) detect the presence and bind to pathogen associated molecular patterns (PAMPs) and damage associated molecular patterns (DAMPs). PAMPs include viral proteins, viral DNA, and viral RNA, whereas DAMPs include products produced from viral associated cell damage and death [34]. The activation of PRRs initiates the production of IFNs, which then bind to their corresponding receptor to initiate a cascade of signaling events, resulting in the activation of IFN stimulated genes (ISGs) to establish an anti-viral effect.

**Figure 1:**
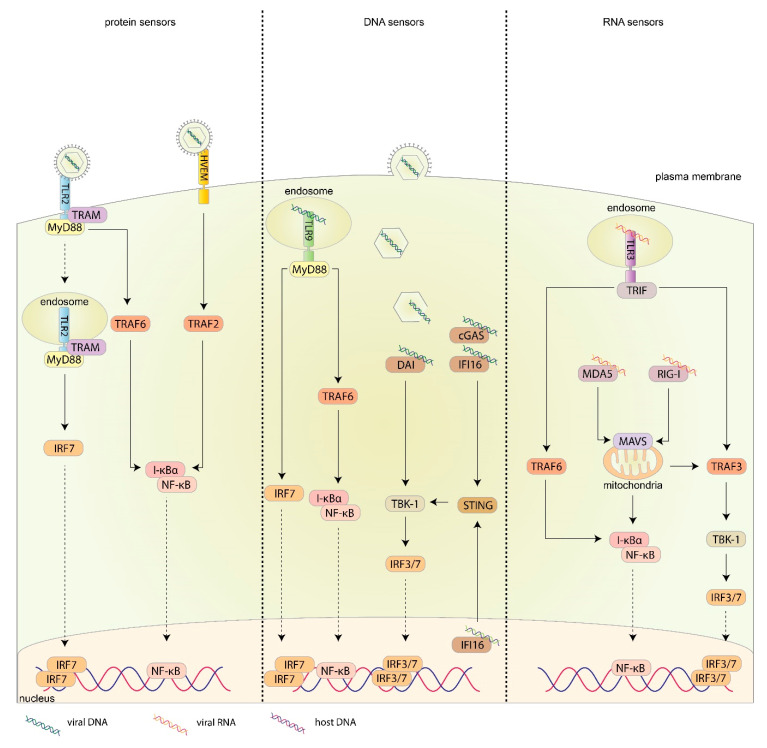
Recognition of HSV-1 by the innate immune system. HSV-1 is recognized by PRRs (pattern recognition receptors) to activate a signaling cascade leading to interferon (IFN) and cytokine production. Viral protein sensors include toll-like receptor (TLR) 2 and herpes virus entry mediator (HVEM). Viral DNA sensors include TLR9, cyclic guanosine monophosphate-adenosine monophosphate synthase (cGAS), IFN inducible protein 16 (IFI16) and DNA-dependent activator of IFN-regulatory factors (DAI). Viral RNA sensors include TLR3, melanoma differentiation-associated protein 5 (MDA5) and retinoic acid-inducible gene I (RIG-I). Solid lines represent downstream activation, dashed lines represent translocation.

IFNs are a class of cytokines that are produced in response to viral infections that interfere with viral replication and are divided into three groups: Type I, II, and III IFNs. In humans, type I IFNs are produced by most cell types and consist of IFNα, β, ϵ, κ, and ω, with IFNα consisting of a further 13 subtypes with varying levels of anti-viral activity [35,36,37]. Type II IFN consists of IFNγ only, which is predominantly produced by natural killer (NK) cells and T cells [35,38]. Type III IFNs are structurally similar to—and are members of—the interleukin (IL) 10 family; however, they activate the same signaling cascade and are functionally similar to type I IFNs [39]. In humans, type III IFNs consist of IFNλ1, IFNλ2, IFNλ3 (also known as IL29, IL28A, and IL28B, respectively), and IFNλ4. The major PRRs activated upon HSV-1 recognition that lead to the production of IFNs are summarized in Figure 1.

### 3.2. Sensing Viral Attachment and Fusion

During viral attachment and entry, there are a number of surface molecules capable of sensing viral proteins and activating an innate immune response. Major viral protein sensors of HSV-1 include the toll-like receptor (TLR) 2, TLR4, and HVEM. The fusion of the HSV-1 envelope to the plasma membrane, even in the absence of a viral capsid (and hence, viral nucleic acids) induces a type I IFN response indicating that the actual binding of HSV-1 to these viral protein sensors is sufficient to elicit an innate immune response [40] (Figure 1).

TLR2 is a member of the TLR family, a class of PRRs that can be found either on the plasma membrane or within the cell cytoplasm associated with endosomes, lysosomes, and the endoplasmic reticulum. There are 12 TLRs identified in mammals; however, the major TLRs implicated in HSV-1 infections are TLR2, TLR3, and TLR9.

TLR2 is a plasma membrane receptor, which upon binding viral glycoproteins, signals through myeloid differentiation factor 88 (MyD88) and tumor necrosis factor receptor associated factor (TRAF) 6 to activate the nuclear factor-κB (NF-κB) pathway (Figure 1). This induces the degradation of I-κBα (an inhibitor of NF-κB), allowing NF-κB to translocate to the nucleus leading to the expression of several pro-inflammatory cytokines and chemokines in several human and mouse cell types, including epithelial, immune, and neuronal cells [34,41,42,43,44]. HSV-1 activation of TLR2 can also signal through TRIF-related adaptor molecule (TRAM) and MyD88 for IFN production (Figure 1). It is hypothesized that HSV-1 binding to TLR2 induces the internalization of the TLR2-TRAM-MyD88 complex and translocation to the endosome. MyD88 then signals through IFN regulatory factor (IRF) 7 for type I IFN production [45].

Soluble viral envelope glycoproteins gH and gL are sufficient to activate TLR2 and it is thought that this is the mechanism by which host cells detect intact virions [46,47]. Soluble viral envelope glycoprotein gB is also able to bind TLR2, but conflicting results have been reported on its ability to activate NF-κB in both cell lines and rabbit cornea [47,48,49]. It is still unclear whether gB as part of an intact virion has the same response. In addition, gH and gL are also able to bind αvβ3-integrin, which works in conjunction with TLR2 to increase the stability of the signaling complex [46]. Interestingly, TLR2 knockout mouse models infected with HSV-1 have a higher survival rate and fewer complications compared to wild type mice [42]. TLR2 deficient mice also have fewer infiltrating macrophages following HSV-1 neuronal infection [50]. This suggests that TLR2 activation and the subsequent immune response generated may be detrimental to the host. Similar models in vitro have shown that suppression of TLR2 (and TLR9) improves host survival by dampening the cytokine response generated [51].

TLR4 is another plasma membrane receptor; however, its role in HSV-1 infection is less defined. TLR4 is activated upon HSV-2 infections in cervical epithelial cells, and signals through both MyD88 and TRIF to activate NF-κB and IRF3/7 for IFN and other cytokine production [52,53,54]. Studies on HSV-1 are more controversial. Mouse models of HSV-1 infections of the central nervous system have shown that TLR4 is not activated during infection [55]; however, TLR4 knockout mice are more susceptible to ocular HSV-1 lesion development, suggesting it may play a role [56]. TLR4 expression in humans is limited, primarily detected in cells of myeloid origin (particularly monocytes), but is absent in other immune cells such as plasmacytoid dendritic cells (pDCs) and Langerhans cells [57,58]. Further investigation is necessary to determine whether TLR4 plays a protective role against HSV-1 infections.

HVEM, a member of the tumor necrosis factor (TNF) receptor superfamily, is one of three receptors that can be utilized during viral fusion; however, it also alerts the immune system to the presence of viral antigens. Being the major entry receptor for corneal epithelial cells, most research into the immune response generated by HVEM has focused on corneal HSV-1 infections. Binding of soluble gD to HVEM signals through TRAF family members (primarily TRAF2) to activate the NF-κB pathway (Figure 1); however, it is still unknown whether gD, as part of the intact virus, has the same effect [59,60]. Interestingly, cytokine production in HSV-1 infected corneas initiated by HVEM can also occur in the absence of gD-HVEM interaction [61]. It is hypothesized that during infection, HVEM also binds several natural ligands expressed by T cells, NK cells, dendritic cells (DCs), and other leukocytes, resulting in an immune response [61]. This is further supported by studies that have shown that, upon HSV-1 infection of the cornea, HVEM induces the infiltration of leukocytes, including macrophages and CD4+ and CD8+ T cells [62]. HVEM also increases the proliferation and responses of regulatory T cells and regulates the levels of cytokine production, including IFNγ in HSV-1 infected corneas [63,64].

### 3.3. Sensing Viral DNA

Once the virus has successfully entered the cell, the naked nucleocapsid is transported to the nucleus where the genome is deposited for viral replication and viral DNA can be sensed. Alternatively, the nucleocapsid can be damaged during transport, releasing viral DNA into the cytoplasm [65]. Viral DNA can also be sensed in endosomes during transport [66]. Major viral DNA sensors of HSV-1 include TLR9 in endosomes, cyclic guanosine monophosphate-adenosine monophosphate synthase (cGAS) in the cytoplasm, IFNγ inducible protein 16 (IFI16) mostly in the nucleus, and DNA dependent activator of IFN-regulatory factors (DAI) (Figure 1).

TLR9 is an endosome-associated transmembrane protein receptor that is activated upon sensing viral DNA containing unmethylated CpG motifs. Similar to TLR2, TLR9 signaling following the recognition of HSV-1 is dependent on MyD88 and TRAF6 resulting in the activation of the NF-κB pathway for downstream cytokine secretion [66]. TLR9 also signals through IRF7 for the production of type I IFNs [66,67,68]. Interestingly, in DCs, type III IFN induction by TLR9 activation is primarily mediated through the NF-κB pathway [69] (Figure 1). While this IFN response is critical in controlling HSV-1 infections, mouse models are able to compensate for a loss of TLR9 and/or MyD88 to amount an adequate IFN response through TLR9/MyD88 independent pathways. This compensation occurs in several cell types including DCs, pDCs, and fibroblasts [44,66,67,70]. Macrophages also have no detectable TLR9 protein or mRNA expression and therefore signal entirely through TLR9-independent pathways [71]. TLR9 also works in conjunction with TLR2 to help control both HSV-1 and HSV-2 infections in mucosal tissue and limit viral replication and dissemination to the brain [72,73].

In order to detect viral DNA, TLR9 must be located within the endosome. The endoplasmic reticulum protein Unc93b is critical in the transport of TLR9 to the endosomal membrane and TLR signaling [74,75]. Any defects to Unc93b results in an impaired IFN response to HSV-1 infections [44,76]. This is evident in patients with Unc93b mutations having a higher susceptibility to HSE [76].

cGAS is a recently discovered DNA sensor that binds to viral dsDNA inducing the synthesis of 2’3’-cGAMP, which then binds to the endoplasmic reticulum associated protein, stimulator of IFN genes (STING), which signals through TANK-binding kinase 1 (TBK1), activating IRF3/7 to induce type I IFN production [77,78]. Alternatively, activation of TBK1 by STING can activate the NF-κB pathway [79] (Figure 1). Recently, it has been shown that β-catenin is an essential component of the cGAS-STING pathway and blocking the nuclear translocation of β-catenin reduces type I IFN production [80]. While cGAS is primarily cytoplasmic, it is partially located in the nucleus of fibroblasts and keratinocytes and there is evidence it plays a role in stabilizing IFI16, another viral DNA sensor (described below) [81,82]. cGAS is also upregulated in astrocytes, but not microglia following HSV-1 infections. This suggests that cGAS may also play a role in controlling HSV-1 infections in the central nervous system, but may be species specific [83,84,85]. This is further supported in mice with cGAS or STING deficiencies having a higher susceptibility to HSE due to uncontrolled HSV-1 replication in neurons [86].

Recently, the deubiquitinating enzyme ubiquitin carboxyl-terminal hydrolase (USP) 27 (USP27X) has been shown to de-ubiquitinate cGAS, increasing its stability. Knockout studies of USP27X in HSV-1 infected murine macrophages exhibit increased viral replication [87]. However, TRAF6, which ubiquitinates cGAS, is also crucial and when TRAF6 is knocked down in murine macrophages and fibroblasts there is also uncontrolled HSV-1 replication [88]. Similar results are also shown in E3 ubiquitin-protein ligase TRIM56 knockout mice, another enzyme that ubiquitinates cGAS [89]. This suggests that the level of cGAS ubiquitination must be tightly regulated for its anti-viral function. Downstream of cGAS activation, USP44 is involved in the deubiquitinating STING following viral infections. USP44 knockout mice are more susceptible to HSV-1 infections indicating the deubiquitination of STING is also critical in this pathway [90].

The specific HSV-1 ligand that is detected by cGAS is currently unknown. It is speculated that perhaps, rather than cGAS directly detecting viral DNA, cGAS rather detects mitochondrial DNA which is released due to herpesvirus infections [34]. Further investigations are necessary to determine the exact mechanisms behind cGAS activation. Interestingly, levels of cGAS expression in human neonates are decreased compared to adults and may contribute to the high susceptibility of newborns developing neonatal herpes [91].

IFI16 is a receptor for HSV-1 dsDNA and its location is cell type dependent and controlled by its level of acetylation [92,93]. IFI16 is capable of sensing viral DNA primarily in the nucleus; however, it can also detect viral DNA in the cytosol and is cell-type specific [92]. In fibroblasts, HSV-1 DNA is detected in the nucleus and is evident from IRF3 localizing to the nuclear periphery upon HSV-1 infection [82]. However, in macrophages there is evidence that IFI16 is capable of detecting HSV-1 DNA in the cytoplasm from degraded viral capsids in addition to viral DNA generated in the nucleus during replication [65]. Regardless of its location, upon binding viral DNA, IFI16 then activates the STING pathway to activate IRF3/7 to induce a type I IFN response [92,94] (Figure 1). This is especially important in epithelial cells and is essential in IRF3/7 activation and IFNα production to control HSV-1 infections. Any knockdown or deletion of IFI16 severely diminishes IFN production in response to HSV-1 infection [95,96,97]. Similar to cGAS, IFI16 does not offer any antiviral protection to microglial, further indicating cell specific functions [85].

IFI16 is also critical in controlling HSV-1 replication in several cell types, including fibroblasts and epithelial cells, independent of IFN activation [98]. IFI16 binds to HSV-1 DNA in the nucleus and induce heterochromatization, silencing viral gene expression, and limiting replication by forming nuclear filamentous structures in conjunction with nuclear domain 10 components [95,98,99,100,101]. In the presence of IFI16, several HSV-1 genes are limited in their expression, including immediate early proteins, infected cell protein (ICP) 0 and ICP4, the early proteins ICP8 and thymidine kinase, and the late proteins gB and pUS11 [98]. IFI16 can also directly bind to the promoter region of type I IFN genes to activate transcription [96].

DAI, also referred to as Z-DNA binding protein, was initially discovered as an unknown molecule that activated type I IFN in response to viral DNA, acting independently of TLR9 [102]. Upon binding HSV-1 DNA, DAI signals through TBK1 and IRF3/7 activating type I IFN production [102] (Figure 1). It can also signal through STING and receptor interacting protein 1 and 3 to activate the NF-κB pathway to secrete inflammatory cytokines, including TNFα [103,104]. While blocking DAI signaling in vitro results in diminished type I IFN production and uncontrolled HSV-1 replication, DAI knockout mice amount a normal immune response, suggesting alternate DNA sensing pathways are able to compensate for this loss [102,105,106]. DAI is upregulated in astrocytes and glial cells in response to HSV-1, and works in conjunction with the RNA sensor retinoic acid inducible gene I (RIG-I) to secrete TNFα, providing further evidence for its protective role against HSV-1 infections [83,104,107].

### 3.4. Sensing Viral RNA

During viral replication, there is the transport and accumulation of double stranded RNA (dsRNA) in the cytoplasm and endosomes of the cell, which can also act as a ligand to activate the innate immune response [108]. Major viral dsRNA sensors of HSV-1 include TLR3 in endosomes, melanoma differentiation-associated protein 5 (MDA5), RIG-I, and protein kinase RNA-activated (PKR) in the cytoplasm. The single stranded RNA (ssRNA) sensors TLR7 and TLR8 may also play a role in sensing HSV-1 RNA in endosomes (Figure 1).

TLR3 is localized in the endosome and recognizes dsRNA produced during viral replication. However, the specific HSV-1 ligand that binds to TLR3 is unknown [109]. Upon activation, TLR3 activates an MyD88-independent signaling cascade through the Toll/IL1 receptor domain, containing adaptor inducing IFNβ (TRIF) and TRAF3, resulting in IRF3/7 translocating to the nucleus, for the production of type I IFNs [110]. TLR3 also signals through TRIF and TRAF6 for NF-κB activation and the production of inflammatory cytokines and chemokines [110] (Figure 1).

Patients with TLR3 deficiencies or mutations are more susceptible to developing HSE, but the importance of TLR3 may be cell-type specific [111,112,113]. Fibroblasts and induced pluripotent stem cells derived from patients with TLR3 deficiencies are susceptible to HSV-1 infection and are unable to control viral replication; however, peripheral blood mononuclear cells and several leukocytes are able to amount a normal IFN response despite TLR3 deficiencies [111,112,114]. Mouse models have shown a similar cell-type specific response. TLR3 deficient astrocytes are susceptible to HSV-2 infections and TLR3 deficient DCs are inefficient in priming CD8+ T cells in response to HSV-1 [115,116]. Neurons and epithelial cell lines that are TLR3 deficient have increased susceptibility to HSV-1 infection [84,117]. However, murine macrophages can compensate for a loss of TLR3 and amount a normal type I IFN response to control HSV-2 infections [70]. Interestingly, TLR3 offers no protection to sensory neurons; the major neuronal cell type infected by HSV-1, unless activated prior to infection [117]. Most recently, it has been hypothesized that the significance of TLR3 may also be species specific [84]. While human fibroblasts are susceptible to HSV-1 infection in the absence of TLR3, murine fibroblasts are able to compensate for this loss [84,111,112,114]. While TLR3 loss can be compensated for in vitro, TLR3 deficiencies and/or mutations in humans are associated with increased susceptibility to HSE [111]. Furthermore, TLR3 transport and signaling is also regulated by Unc93b further contributing to the susceptibility to those with Unc93b mutations to HSE [76,118]. It is clear further research is required to determine the specific role of TLR3 in controlling herpesvirus infections.

RIG-I-like receptors are another class of viral dsRNA sensors and include MDA5 and RIG-I. Upon activation, MDA5 and RIG-I bind to mitochondrial antiviral signaling protein (MAVS), which activates the IRF3/7 and NF-κB pathways, leading to type I and type III IFN production, amongst other cytokines [119] (Figure 1). MAVS is also located in peroxisomes and its activation results in type III, but not type I, IFN production [120]. MDA5 senses long dsRNA (>1000 base pairs), whereas RIG-I senses short dsRNA (<300 base pairs) with a 5’-triphosphate group [119]. MAVS has been identified as a critical signaling molecule in activating the IFN response in macrophages and fibroblasts but not conventional DCs [121,122,123].

MDA5 knockdown in human macrophages results in decreased production of IFNβ and IFNλ1 following HSV-1 and HSV-2 infection; however, this was not observed in murine macrophages, indicating a possible species difference [121,122]. MDA5 is also upregulated in astrocytes following HSV-1 infections where it is thought it play a protective role [83]. While knockdown studies have shown that RIG-I is not essential in amounting a type I IFN response against both HSV-1 and HSV-2 infections in macrophages, it has been shown to work synergistically with MDA5 in sensing cytosolic viral RNA to induce type I IFNs [121,122,124]. While recent work has attempted to narrow down the type of viral and/or host RNA that MDA5 and RIG-I bind to, the specific herpesvirus ligands that are sensed prior to MAVS activation are unknown [125].

Interestingly, RIG-I is also capable of detecting cytosolic DNA by using an intermediary enzyme, RNA polymerase III (RNApol III). RNApol III detects foreign cytosolic DNA and converts it into RNA with a 5’-triphosphate group that is detectable by RIG-I, eliciting a type I IFN response [126]. While patients with mutations in RNApol III are more susceptible to infections with another alphaherpesvirus, VZV, its role in HSV-1 infections is less defined [127]. RNApol III is capable of detecting HSV-1 DNA in human macrophages, but it does not seem to play a role in activating an IFN response [121]. Further work is necessary to elucidate the role of RNApol III in other cell types during HSV-1 infections.

PKR is a cytoplasmic protein capable of detecting viral dsRNA and interacts with several PRRs complicating its role in viral infections. Its major role is to induce the phosphorylation of eIF-2α, a critical enzyme in protein synthesis to inhibit the translation of viral mRNA [128,129]. Binding of PKR to dsRNA induces a signaling cascade activating NF-κB resulting in type I IFN production, as well as other cytokines [130]. PKR is also activated by a number of TLRs, including TLR3 and TLR4, further enhancing this effect [131,132]. PKR is also crucial in MDA5, but not RIG-I, activation of MAVS for type I IFN production [133]. However, it should be noted that while PKR is an efficient anti-viral agent, HSV-1 effectively blocks its effects and these anti-viral effects are only observed in ICP34.5 null HSV-1 mutants [134].

TLR7 and TLR8 are also localized in the endosome but recognize ssRNA. Little is known on their role in activating an immune response against HSV-1; however, recently it has been shown that TLR7 and TLR8 mRNA and protein levels are temporarily increased in astrocytes following HSV-1 infection [83]. Similarly, TLR7 is upregulated in HSV-1 infected corneal epithelial cells [135]. While this suggests that both may play a protective role against HSV-1 infections, further research is necessary to confirm this.

## 4. The Interferon Response against HSV

### 4.1. IFN Production in Herpes Lesions

**Figure 2:**
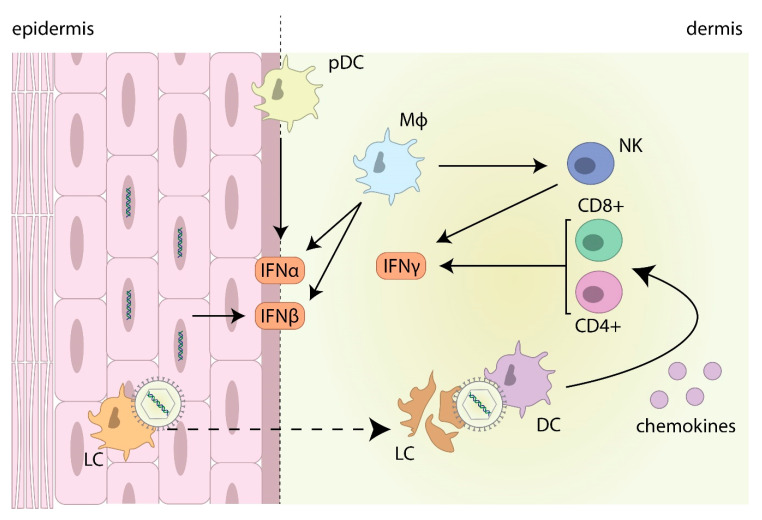
Interferon production in herpes lesions. HSV-1 infected keratinocytes secrete IFNβ, and infiltrating plasmacytoid dendritic cells (pDCs) secrete primarily IFNα. Infiltrating macrophages (Mϕ) contribute to IFNα and IFNβ production, as well as secreting IL12, to activate natural killer (NK) cells to secrete IFNγ. HSV-1 infected Langerhans cells (LCs) migrate to the dermis where they undergo apoptosis, and HSV-1 antigens are taken up by dermal dendritic cells (DCs) to attract CD4+ and CD8+ T cells which also secrete IFNγ.

In herpes lesions, infected keratinocytes are a major source of IFNβ, and pDCs infiltrating into the dermis are a major source of both IFNα and IFNβ [136,137,138] (Figure 2). While pDCs are usually absent in healthy skin, they infiltrate the dermis upon infection and interact with other immune cells in order to control viral replication [139]. It has recently been shown that in vitro, IFNκ is also secreted by human keratinocytes and blocking IFNκ expression enhances HSV-1 replication, indicating it may also play a role in controlling HSV-1 in herpes lesions [140]. Secreted type I IFN, among other cytokines, including IL12, IL15, and IL18, activate NK cells, which produce IFNγ, which plays an important protective role against both HSV-1 and HSV-2 infections [35,141,142]. CD4+ and CD8+ T cells also infiltrate the dermis contributing to the production of IFNγ [143,144,145] (Figure 2). It is hypothesized that this partly occurs due to HSV-1 infected Langerhans cells migrating from the epidermis into the dermis, where they undergo apoptosis and are taken up by dermal DCs, which secrete chemokines and act as the major antigen presenting cell to CD8+ T cells in the draining lymph node and resident T cells in the mucosa [146] (Figure 2). Recent evidence also suggests that dermal DCs and conventional DCs also work together to generate an adequate CD4+ T cell response [147]. Studies on HSV-2 infections have shown that a subset of CD8+ T cells, specific for HSV, persists in the mucosa long after viral clearance from the lesion. It is hypothesized that they act as resident T cells involved in immune surveillance to control herpesvirus infections [148,149]. Infiltrating macrophages are also involved in the early production of IFNα and IFNβ, and at later stages of infection secrete IL12 to further enhance the production of IFNγ by NK cells [150]. The combination of IFNγ and IFNα/β secreted at the site of infection also work synergistically both in vitro and in vivo to limit HSV-1 replication [151,152]. The cytokine IL36 has recently been identified as an enhancer of the type I IFN response in herpes lesions, upregulating the expression of IFNAR on keratinocytes and the subsequent activation of signal transducer and activator of transcription (STAT) 1 and STAT2 [153].

It is also hypothesized that intercellular communication occurs between infected and non-infected cells through extracellular vesicles. Extracellular vesicles from HSV-1 infected cells contain host and viral factors, such as STING, and when uninfected cells are treated with these extracellular vesicles, there is the induction of several ISGs [154]. While this has only been shown in vitro, it is hypothesized that in herpes lesions, intercellular communication through extracellular vesicles is important in priming and alerting uninfected cells of viral infection and controlling dissemination.

### 4.2. Canonical vs. Non-Canonical IFN Pathways

**Figure 3:**
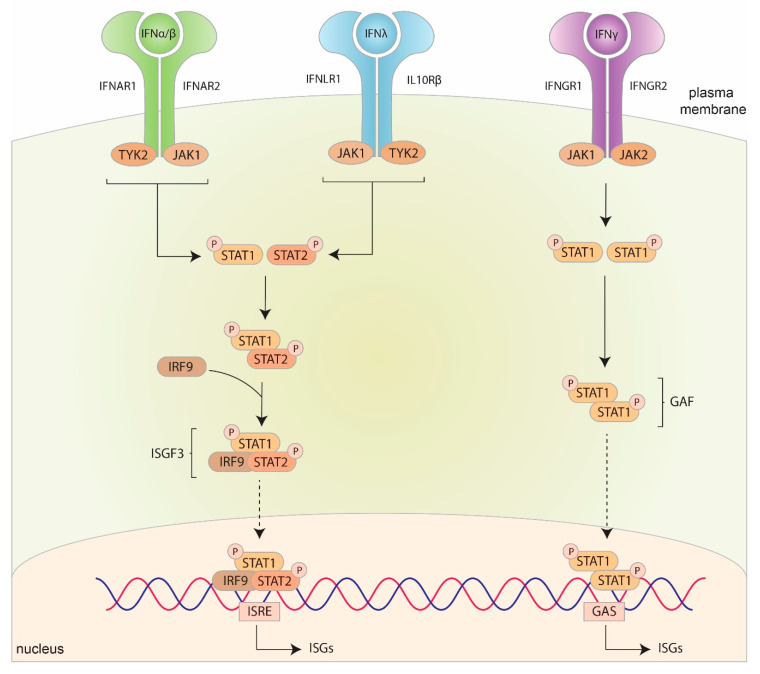
Canonical signaling pathways of interferon (IFN). The binding of type I IFNs to interferon-α receptor (IFNAR) and type III IFNs to interferon-λ receptor (IFNLR) results in the activation of Janus kinase 1 (JAK1) and tyrosine kinase 2 (TYK2) resulting in the phosphorylation of signal transducers and activators of transcription (STAT) 1 and 2. STAT1 and STAT2 interact with interferon regulatory factor (IRF) 9 to form the complex IFN-stimulated gene factor 3 (ISGF3), which translocates to the nucleus to bind to interferon-stimulated response element (ISRE) to induce the expression of ISGs. The binding of type II IFN to interferon-γ receptor (IFNGR) results in the activation of JAK1 and JAK2 resulting in the phosphorylation of STAT1. STAT1 then forms a STAT1-STAT1 homodimer, also known as gamma interferon activation factor (GAF), which translocates to the nucleus to bind to gamma interferon activated sites (GAS) to induce the expression of ISGs. Solid lines represent downstream activation, dashed lines represent translocation.

The classical—or ‘canonical’—pathway activated by IFNs is the Janus Activated Kinase (JAK)/STAT pathway, where IFNs activate—or phosphorylate—STAT1 and/or STAT2 for downstream signaling (Figure 3).

Each subunit of the IFN receptors associate with different members of the JAK family. Type I IFNs bind to the IFNα receptor (IFNAR), composed of two subunits, IFNAR1 and IFNAR2, which associate with tyrosine kinase 2 (TYK2) and JAK1, respectively. Type II IFN binds to the IFNγ receptor (IFNGR), composed of IFNGR1 and IFNGR2, which associate with JAK1 and JAK2, respectively. Type III IFNs bind to the IFNλ receptor (IFNLR), composed IFNLR1 (also known as IL28Rα) and IL10Rβ [38]. IL10Rβ is widely expressed and is shared with other receptors that bind members of the IL10 family. However, IFNLR1 is specific for IFNλ and is mainly detected in epithelial and neuronal cells [155,156]. IFNLR1 and IL10Rβ associate with JAK1 and TYK2, respectively [35,38] (Figure 3).

Upon binding their respective receptor, a cascade of events occurs. IFNα/β binding to IFNAR and IFNλ binding to IFNLR induces the dimerization of the two receptor subunits resulting in the autophosphorylation and activation of TYK2 and JAK1. This then leads to the tyrosine phosphorylation of STAT1 and STAT2, which form a heterodimer. This heterodimer, along with IRF9 then form a complex known as ISG Factor 3 (ISGF3). ISGF3 translocates to the nucleus and binds to IFN-stimulated response elements (ISRE) resulting in the transcription of ISGs [157,158,159,160] (Figure 3). Bclaf1 (bcl-2-associated transcription factor) has recently been identified as an important regulator during this process and is involved in the phosphorylation of STAT1 and STAT2 as well as facilitating the binding of ISGF3 to ISRE [161]. Impairing the function of Bclaf1 results in decreased IFNα signaling [161].

The binding of IFNγ to IFNGR similarly results in the dimerization of the two receptor subunits resulting in the autophosphorylation and activation of JAK1 and JAK2. This then leads to the tyrosine phosphorylation of STAT1, which form STAT1-STAT1 homodimers (also referred to as gamma IFN activation factor (GAF)). GAF then translocates to the nucleus and binds to gamma IFN activated sites (GAS), resulting in the transcription of alternative ISGs [162] (Figure 3).

The canonical pathways are unable to explain how different type I IFNs bind to the same receptor and activate the same ISGF3 complex, but result in different ISG production and varying levels of anti-viral activity [36,163]. It also fails to address how, even though type III IFNs activate the same pathway as type I IFNs, type III IFNs are only capable of activating a subset of the ISGs activated by type I IFNs, and at different levels and kinetics [164,165]. This can partly be explained by type I IFNs binding with different affinities to IFNAR, with IFNα1 binding with the lowest affinity [166,167] and IFNβ with the highest affinity [168]. However, this does not give a complete explanation for this observation. Therefore, it is believed there are much more complex interactions taking place through ‘non-canonical’ pathways, for all three types of IFN. These non-canonical pathways may involve un-phosphorylated STATs capable of activating other subsets of genes, endocytosis of the entire receptor complex upon IFN activation, and the formation of alternate transcriptional complexes other than ISGF3 and GAF [11,169,170]. For example, Type I and III IFNs binding to their respective receptors are also capable of forming the GAF complex to activate GAS. Additionally, other STAT molecules are also activated by both type I and type III IFNs and can result in the formation of homo and hetero-dimers of STAT3, STAT4, STAT5, and STAT6 adding a further level of complexity to these pathways [38]. For an in-depth review of the type I and III IFN signaling cascade, see [38] and for the type II IFN signaling cascade see [162].

### 4.3. ISG Induction in Response to HSV

A number of the PRRs described above are also classified as ISGs and while they are always present at baseline levels in cells, they are upregulated in response to IFN. This is important in increasing the ability of host cells to recognize PAMPs to enhance the IFN response and the overall antiviral state. PRRs that recognize HSV-1 that are upregulated by IFNs include TLR2, TLR3, TLR4, TLR7, cGAS, IFI16, MDA5, RIGI-I, and PKR [71,129,171,172,173,174,175,176]. In addition to these PRRs, a number of downstream adaptor molecules activated by PRRs are also upregulated by IFNα including MyD88, TRIF, TBK1, IRF7, and receptor interacting protein 1 [171].

While there are hundreds of ISGs that are induced by IFN, HSV-1 has evolved mechanisms to counteract or diminish their anti-viral effects (outlined in Section 5). Anti-viral ISGs that are known to have an effect on wild type HSV-1 include myxovirus resistance protein (Mx) A, MxB, tetherin, ISG15, PKR, the 2′,5′-oligoadenylate synthase (OAS)/RNase L pathway and IFNγ induced protein 10 (CXCL10). The effects of these ISGs are summarized in Table 1.

### 4.4. Role of IFN in Controlling HSV-1 Infections

The role of IFNs in controlling HSV-1 infections is well described in the literature. Mouse models unable to amount a sufficient type I IFN response, either through inadequate IFNα/β levels [188,189] or IFNAR knockouts [190,191] are unable to adequately control HSV-1 and 2 replication resulting in widespread infection. Similarly, knockout models of IFNγ and IFNGR are also unable to control HSV-1 replication [192]. Interestingly, one study showed loss of the type II IFN response, through knockdown of IFNGR in a mouse model, had no effect on HSV-1 replication [190]; however, dual knockout of both IFNAR and IFNGR results in uncontrollable dissemination and death [190]. IRF3 and IRF7 are also both critical in the control of herpes infection, and the progression of HSE. IRF3 deficiencies in humans have been associated with HSE, believed to be due to the inability to amount an adequate type I IFN response [193]. This was also observed in IRF3 knockout mouse models [157]. However, it was recently shown that IFN production is only impaired early on during infection. When type I IFN levels were assessed in IRF3 or IRF7 knockout mouse models at later stages of infection (5 days post infection), significantly higher levels of IFN were produced compared to wild type [194]. This was also associated with increased mortality in IRF3 and IRF7 knockout mice compared to wild type [194]. This indicates the critical role that IRF3 and IRF7 play in not only activating the type I IFN response at early stages of infection, but also in controlling these IFN levels to prevent neurotoxicity.

While originally thought to be immune privilege, neurons are also capable of responding to IFN to control HSV-1 infections at all stages of the HSV-1 life cycle. HSV-1 infected TG neurons obtained from mice are also capable of producing their own type I IFN [41]. IFNβ treatment of rat sensory neurons results in the phosphorylation of STAT1 and transcription of several ISGs, including ISG15 [195,196]. In addition, IFNβ and IFNγ treatment of axons induce changes in the proteomic profile of these axons [195]. Pretreating these axons with either IFNβ or IFNγ reduces HSV-1 retrograde transport to the cell body, without affecting the retrograde or anterograde transport of other cargo (e.g., lysosomes) [195,196]. In the cell body, all three types of IFNs can control and limit HSV-1 replication in rat neurons [197,198]. Following anterograde transport, IFNα and IFNγ inhibit the replication and spread of HSV-1 in human skin explants following transmission from DRG axons [152]. IFNα and IFNγ also work synergistically to maximize this inhibition [152]. This was assumed to be only an IFN effect on epithelial cells but an effect on axons may also contribute. IFNγ also plays a role in limiting reactivation of HSV-1 in neurons. Several studies in human and mouse TG neurons have shown that HSV-1 specific CD8+ T cells within the ganglia secrete IFNγ and target viral proteins to control reactivation [199,200].

## 5. HSV-1 Evasion Response against the Innate Immune System

**Figure 4:**
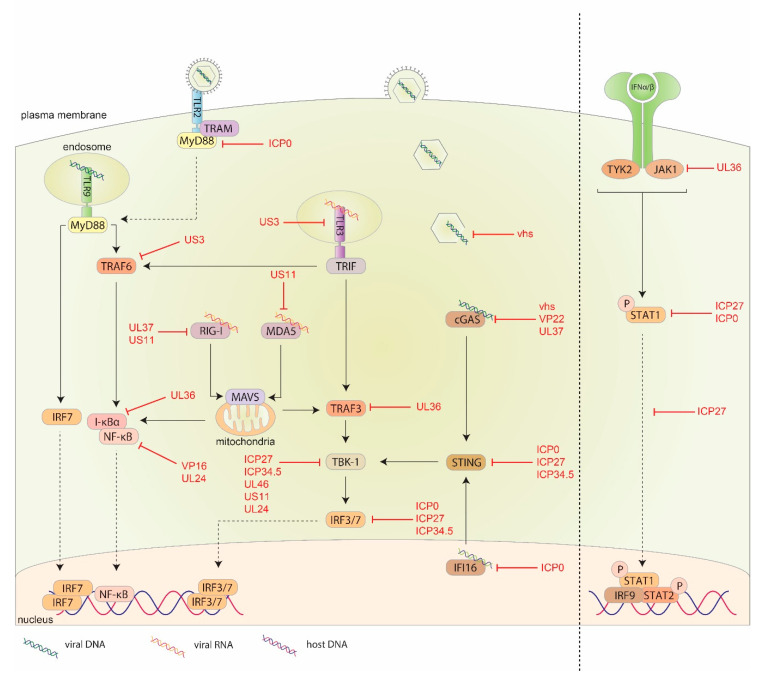
HSV-1 evasion mechanisms to downregulate the host innate immune response. Multiple steps in the downstream signaling pathways following PRR activation are targeted by HSV-1 proteins to limit detection by the innate immune system. HSV-1 can directly inhibit the DNA sensors cGAS and IFI16, and the RNA sensors TLR3, RIG-I and MDA5 as well the accumulation of viral DNA in the cytoplasm. HSV-1 also inhibits downstream signaling molecules including MyD88, TRAF3, TRAF6, I-κBα, STING, and TBK-1 to prevent the nuclear translocation of IRF3/7 and NF-κB, preventing IFN and cytokine production. HSV-1 also inhibits IRF3/7 and NF-κB directly. HSV-1 has also evolved to inhibit the IFN signaling pathway directly, inhibiting JAK1 phosphorylation as well as STAT1 phosphorylation and its translocation to the nucleus, downregulating ISG production. Solid lines represent downstream activation, dashed lines represent translocation.

While the immune system has developed several defense mechanisms against invading pathogens, HSV-1 has evolved its own evasion mechanisms allowing it to successfully infect host cells and establish latency. HSV-1 encodes for several proteins that block IFN induction and signaling pathways, inhibit ISG transcription and interfere with the antiviral functions of ISGs (Figure 4). The major viral proteins involved in evading the host innate immunity are described below and summarized in Table 2.

### 5.1. HSV-1 pUS3

pUS3 plays a role in downregulating the type I IFN response induced by TLR3 activation. Human monocytes infected with pUS3 null HSV-1 showed higher levels of TLR3 and IRF3 expression as well as increased production of IFNβ and MxA transcription compared to wild type HSV-1 [201]. This suggests pUS3 acts to downregulate TLR3 and the subsequent activation of IRF3 to dampen the type I IFN response (Figure 4). pUS3 also promotes the proteasomal degradation of bclaf2 and as a result IFNα signaling and ISG induction is diminished. This was observed in HEp-2 cells infected with pUS3 null mutant HSV-1 that were unable to induce the degradation of bclaf2 compared to wild type virus [161].

pUS3 is also involved in inhibiting the nuclear translocation of NF-κB following activation of TLR2 by blocking the ubiquitination of TRAF6 [202] (Figure 4). This was observed in both human HEK293T cells and murine macrophages [202]. pUS3, in conjunction with pUL13 also dampens the IFNγ response by inhibiting the phosphorylation of IFNGR1, a requirement for complete activation of the IFNGR receptor [203]. Recent evidence has shown that pUS3 also inhibits the actions of β-catenin following activation of the cGAS-STING pathway by blocking the nuclear translocation of β-catenin in mouse fibroblasts, further dampening the type I IFN response [80].

### 5.2. HSV-1 Virion Host Shutoff Protein (vhs)

vhs allows HSV-1 to evade the innate immune system through several mechanisms. vhs has RNase activity to promote the selective degradation of host cell mRNA, resulting in the inhibition of ISG expression, and prioritizes the transcription of viral mRNAs [204,205]. One important ISG degraded by vhs is viperin, an ISG involved in inhibiting viral replication. While several viruses are known to induce viperin expression, HSV-1 effectively degrades viperin RNA in vitro [206]. While vhs does not degrade all viperin mRNA, it is believed other viral proteins (e.g., gD) inhibit what remains [207]. vhs also reduces the accumulation of viral dsRNA in host cells (Figure 4). Human foreskin fibroblasts and HeLa cells infected with vhs null HSV-1 had higher levels of dsRNA accumulating in the cells compared to wild type virus [208]. vhs plays an important role in regulating the levels of dsRNA available for RNA sensors to detect, limiting the innate immune response.

vhs is also involved in inhibiting the phosphorylation of eIF2α, essentially nullifying the effects of PKR to allow viral transcription. This is evident from studies conducted using vhs null HSV-1 and HSV-2 mutants showing increased phosphorylation of eIF2α in mouse fibroblasts compared to wild type virus [209].

vhs null virus also induce higher levels of IFNβ and ISG production compared to wild type virus suggesting vhs plays a role in dampening IFN production, and ISGs such as tetherin [180,209]. This is further supported by HEK293T cells transfected with vhs showed that vhs downregulates the production of cGAS [210] (Figure 4).

### 5.3. HSV-1 Viral Protein 16 (VP16)

VP16 binds to IRF3 preventing it from binding to DNA in the nucleus, reducing the transcription of type I IFNs [211] (Figure 4). This was observed in HEK293T cells transfected with VP16. IRF3 phosphorylation and translocation to the nucleus is unaffected by VP16, but its ability to bind to CREB-binding protein (CBP), a co-activator of IRF3 required for binding to the IFNβ promotor region, is inhibited, resulting in decreased IFNβ production and subsequent activation of ISRE [211]. This was also confirmed by pulldown assays showing VP16 from wild type HSV-1 interacts with IRF3, but not IRF7 [211]. VP16 is also implicating in inhibiting the NF-κB pathway by interacting with NF-κB subunits and blocking the activation of the NF-κB promoter region [211]. VP16 is also involved in inhibiting peroxisomal MAVS. HEK293 cells transfected to produce VP16 showed decreased activation of peroxisomal MAVS and ISG induction [212].

### 5.4. HSV-1 ICP27

ICP27 inhibits the type I IFN response by inhibiting the phosphorylation of STAT1, hence directly inhibiting the canonical pathway of type I IFNs (Figure 4). ICP27 null HSV-1 induces high levels of STAT1 phosphorylation compared to wild type virus in Vero cells, following IFNα treatment [213]. ICP27 null HSV-1 also induced higher levels of phosphorylated STAT1 accumulating in the nucleus suggesting ICP27 also plays a role in blocking nuclear translocation of phosphorylated STAT1 [213]. When Vero cells are transfected with ICP27 and stimulated with IFNα, phosphorylated STAT1 localization to the nucleus is completely blocked, and is due to the secretion of an unknown, heat-stable factor that specifically targets the type I IFN response [214]. No changes to STAT1 phosphorylation levels were observed in ICP27 transfected Vero cells following IFNγ treatment, indicating the selectivity of this unknown protein to the type I IFN pathway [214].

ICP27 further inhibits the production of type I IFNs by interfering with the cGAS-STING pathway. Several cell lines including human and mouse macrophages and human fibroblasts infected with ICP27 null HSV-1 induced higher levels of IFNα production compared to wild type virus and this increased IFNα production was dependent on the cGAS-STING pathway [215]. ICP27 interacts with TBK1 and STING to prevent the phosphorylation of IRF3 and subsequent translocation to the nucleus [215] (Figure 4). ICP27 also works in conjunction with vhs to reduce host cell mRNA levels [216].

### 5.5. HSV-1 ICP0

ICP0 works to inhibit the innate immune system through several mechanisms. ICP0 inhibits the activation of IRF3 and IRF7 resulting in decreased ISG induction (Figure 4). Human lung fibroblasts transfected to produce ICP0 had decreased IRF3 and IRF7 activation and subsequent production of ISGs [217]. Similarly, when infected with ICP0 null HSV-1, there is evidence of increased IRF3 dimerization [218]. This is further supported by foreskin fibroblasts infected with mutant HSV-1 capable of expressing ICP0 (but not other immediate early genes ICP4, ICP22, ICP27, and ICP47) inducing the degradation of IFI16 and sequestering IRF3 [94,219]. However, the degradation of IFI16 is cell type dependent, evident in foreskin fibroblasts and immortalized keratinocytes, but not in HeLa cells or osteosarcoma epithelial cells [220]. ICP0 also seems to play a role in the STING pathway. ICP0 null HSV-1 has impaired STING pathway activation in osteosarcoma cell line, further inhibiting the activation of IRF3 [221].

Similar to ICP27 null HSV-1, ICP0 null HSV-1 induced higher levels of STAT1 phosphorylation compared to wild type virus, but not to the same extent as ICP27 (Figure 4). Therefore ICP0 may work in conjunction with ICP27 to inhibit the phosphorylation of STAT1 and hence dampening the type I IFN response [213].

ICP0 also inhibits TLR2 signaling and the NF-κB pathway (Figure 4). HEK293 cells transfected with ICP0 had limited TLR2 activation due to ICP0 inhibiting the activation, and reducing the levels of MyD88 and Mal (MyD88 adaptor-like protein), critical signaling molecules required for TLR2 activation of the NF-κB pathway [222].

### 5.6. HSV-1 ICP34.5

Similar to vhs, ICP34.5 inhibits the function of PKR by dephosphorylating eIF2α to prevent host mRNA translation. HeLa cells infected with ICP34.5 null HSV-1 had decreased eIF2α phosphatase activity compared to wild type virus [134].

ICP34.5 inhibits the phosphorylation of IRF3, preventing its translocation to the nucleus and subsequent type I IFN production (Figure 4). How this is achieved is still not completely understood. Earlier studies in HEK293T cells transfected with ICP34.5 showed an interaction with TBK1 and it was hypothesized that this inhibited the phosphorylation and nuclear translocation of IRF3 [223]. However, later studies showed that ICP34.5 only binds to TBK1 when overexpressed and not to endogenously expressed TBK1 [224]. It is hypothesized that ICP34.5 may use an intermediary protein, such as beclin-1, to indirectly bind to TBK1; however, this is yet to be shown [224]. ICP34.5 also inhibits the activation of STING as observed in human fibroblasts infected with ICP34.5 null HSV-1 inducing higher levels of STING phosphorylation compared to wild type virus [225]. Given that STING activation is an important upstream event of IRF3 phosphorylation, this provides further clarification as to how IFR3 translocation is inhibited.

ICP34.5 also plays a role in neurons, counteracting axonal IFN signaling. ICP34.5 null HSV-1 had impaired retrograde transport compared to wild type virus following axonal treatment by IFNβ [196]. Interestingly, while the IFNβ treatment enhanced this impairment, ICP34.5 null HSV-1 retrograde transport was slightly impaired even in the absence of IFNβ suggesting ICP34.5 also plays an important role in virus transport [196].

### 5.7. Other HSV-1 Proteins Involved in Suppressing IFN

In addition to the major viral protein previously described, other viral proteins have shown to have minor roles in inhibiting the IFN response (Figure 4). These are summarized in Table 3.

## 6. Conclusions

In summary, HSV-1 has evolved several mechanisms to downregulate and inhibit recognition by the innate immune system and the subsequent induction of the IFN response. The ability of the virus to successfully evade immune detection is critical to establish lifelong latency in the host. However, it is clear that the host immune response plays an important role in limiting viral replication and spread following reactivation. This is evident from patients suffering from host immune deficiencies (such as mutations in TLR3 and Unc93b), which can lead to dissemination of the virus throughout the body. This is of concern when HSV-1 disseminates to the central nervous system, resulting in high morbidity and mortality.

Elucidation of the mechanisms of IFN induction, action, and evasion related to HSV are important for guiding future immunotherapy strategies against reactivated HSV. However, the multiple redundant mechanisms of viral immune evasion will prove challenging for the development of these treatments for control of recurrent herpes.

## Figures and Tables

**Table 1:** ISGs known to limit HSV-1 infections.

ISG	Function	Effect on HSV-1	Reference
MxA	GPTase protein which limits genome replication and viral capsid transport	MxA induced by IFNα reduces HSV-1 replication in human fibroblasts. pDCs infiltrating into the dermis of herpes lesions may be involved in stimulating the production of MxA by surrounding cells	[139,177]
MxB	GPTase protein which blocks viral DNA entering the nucleus	Inhibits HSV-1 replication in several human epithelial and neuronal cell lines	[178,179]
Tetherin	Membrane glycoprotein that ‘tethers’ viral particles to the cellular membrane, preventing release	Reduced HSV-1 spread in human epithelial cells, monkey fibroblasts and mouse cornea following upregulation of tetherin. This reduction is enhanced in virion host shutoff factor (vhs) null HSV-1	[180,181,182]
ISG15	Ubiquitin-like protein which conjugates to proteins (>100 known) to induce post-translational modifications	ISG15 knockout mice are unable to control HSV-1 infections and are associated with the formation of autophagic clusters of TG neurons	[183,184]
PKR	Phosphorylates eIF-2α upon binding viral dsRNA to limit mRNA translation	Mouse TG neurons with PKR or RNase L knockouts have increased HSV-1 replication compared to wild type TG neurons in the presence of IFNβ	[185]
OAS/RNase L	OAS synthesizes 2′,5′-oligoadenylate that binds to RNase L, which then cleaves mRNA		
CXCL10	IFNγ induced protein involved in recruiting NK and CD8+ T cells to infection sites	CXCL10 null mice infected with HSV-1 and 2 have reduced numbers of NK and CD8+ T cells at the site of infection and increased viral replication	[186,187]

**Table 2:** Major viral proteins involved in immune evasion.

Viral Protein	Role in Suppressing the Innate Immune Response	Reference
pUS3	Inhibits TLR3 expression	[201]
	Induces the degradation of bclaf2	[163]
	Inhibits the ubiquitination of TRAF6	[204]
	Inhibits the phosphorylation of the IFNGR1 subunit	[205]
	Inhibits the nuclear translocation of β-catenin	[84]
vhs	Degrades host mRNA to inhibit ISG translation	[206,207]
	Prevents accumulation of viral DNA in cell cytoplasm	[210]
	Inhibits the phosphorylation of eIF2α	[211]
	Downregulates cGAS production	[212]
VP16	Binds to IRF3 and prevents its interaction with CBP	[213]
	Blocks NF-κB binding to promoter region	[213]
	Inhibits peroxisomal MAVS activation	[214]
ICP27	Inhibits the phosphorylation and nuclear translocation of STAT1	[215]
	Inhibits TBK1 and STING downstream signaling	[217]
	Degrades host mRNA to inhibit ISG translation	[218]
ICP0	Inhibits IRF3 and IRF7 activation	[219,220]
	Degrades IFI16	[98,221,222]
	Inhibits STING activation	[223]
	Inhibits the phosphorylation of STAT1	[215]
	Reduces levels of MyD88 downstream from TLR2 activation	[224]
ICP34.5	Inhibits the phosphorylation of eIF2α	[138]
	Indirectly binds to and inhibits TBK1	[225,226]
	Inhibits STING activation	[227]

**Table 3:** Minor viral proteins involved in immune evasion.

HSV-1 Proteins	Role in Suppressing the Innate Immune Response	Reference
pUL13	Induces the expression of suppressor of cytokine signaling 1 and 3, which act to suppress IFN production	[226]
pUL42	Inhibits the phosphorylation of IRF3	[227]
pUL24	Prevent the nuclear translocation of NF-κB subunits	[228]
	Inhibits the interaction between TBK1 and IRF3, preventing the dimerization of IRF3 and its translocation to the nucleus	[229]
pUL46	Inhibits TBK1 activation and IRF3 translocation to the nucleus	[230]
VP22	Inhibits cGAS from synthesizing cGAMP	[231]
pUL36	Deubiquitinates TRAF3 impairing downstream signaling, inhibiting IRF3 dimerization	[232]
	Deubiquitinates I-κBα, inhibiting its degradation. This results in sequestering NF-κB in the cytoplasm and preventing its translocation to the nucleus	[233]
	Binds to IFNAR2 and prevents the phosphorylation of JAK1	[234]
pUL37	Deamidates RIG-I and cGAS inhibiting the ability to sense dsRNA and dsDNA respectively	[235,236]
pUS11	Interacts with both MDA5 and RIG-I blocking their interaction with MAVS	[237]
	Inhibits TBK1	[238]

## Abbreviations

Bclaf1	bcl-2-associated transcription factor
cGAS	Cyclic guanosine monophosphate-adenosine monophosphate synthase
CXCL10	Interferon gamma induced protein 10
DAI	DNA-dependent activator of IFN-regulatory factors
DAMP	Damage associated molecular pattern
DC	Dendritic cell
dsDNA	Double stranded DNA
dsRNA	Double stranded RNA
DRG	Dorsal root ganglia
GAF	Gamma interferon activation factor
GAS	Gamma interferon activated sites
gB	HSV-1 envelope glycoprotein B
gC	HSV-1 envelope glycoprotein C
gD	HSV-1 envelope glycoprotein D
gH/gL	HSV-1 envelope glycoprotein H/L heterodimer
HSE	Herpes encephalitis
HSV	Herpes simplex virus
HVEM	Herpes virus entry mediator
ICP0	HSV-1 infected cell protein 0
ICP27	HSV-1 infected cell protein 27
ICP34.5	HSV-1 infected cell protein 34.5
IFI16	Interferon inducible protein 16
IL	Interleukin
IFN	Interferon
IFNAR	Interferon alpha receptor
IFNGR	Interferon gamma receptor
IFNLR	Interferon lambda receptor
ISGF3	Interferon-stimulated gene factor 3
ISRE	Interferon-stimulated response element
IRF	Interferon regulatory factor
ISG	Interferon-stimulated gene
JAK	Janus-activated kinase
LAT	Latency associated transcript
MAVS	Mitochondria antiviral signaling protein
MDA5	Melanoma differentiation-associated protein 5
Mx	Myxovirus resistance protein
MyD88	Myeloid differentiation factor 88
NF-κB	Nuclear factor kappa B
NK	Natural killer
OAS	2′,5′-oligoadenylate synthase
PAMP	Pathogen associated molecular pattern
pDC	Plasmacytoid dendritic cell
PRR	Pattern recognition receptor
RIG-I	Retinoic acid-inducible gene I
RNApol III	RNA polymerase III
ssRNA	Single stranded RNA
STAT	Signal transducer and activator of transcription
STING	Stimulator of interferon genes
TBK	TANK-binding kinase
TG	Trigeminal ganglia
TLR	Toll-like receptor
TNF	Tumor necrosis factor
TRAF	Tumour necrosis factor associated factor
TRAM	TRIF related adaptor molecule
TRIF	Toll/IL1 receptor domain containing adaptor inducing interferon beta
TYK	Tyrosine kinase
pUL13	HSV-1 protein unique long 13
pUL24	HSV-1 protein unique long 24
pUL36	HSV-1 protein unique long 36
pUL37	HSV-1 protein unique long 37
pUL42	HSV-1 protein unique long 42
pUL46	HSV-1 tegument protein UL46
pUS3	HSV-1 unique short 3
pUS11	HSV-1 unique short 11
USP	Ubiquitin carboxyl-terminal hydrolase
vhs	HSV-1 virion host shutoff protein
VP16	HSV-1 viral protein 16
VP22	HSV-1 viral protein 22
VZV	Varicella zoster virus
